# Epidemiology and history of knee injury and its impact on activity limitation among football premier league professional referees

**DOI:** 10.5249/jivr.v10i1.963

**Published:** 2018-01

**Authors:** Hamid Mahdavi Mohtasham, Shahnaz Shahrbanian, Fatemeh Khoshroo

**Affiliations:** ^*a*^Bone, Joint and Related Tissue Research Center, Shahid Beheshti University of Medical Sciences, ‎Tehran, ‎Iran.; ^*b*^Department of Sport Rehabilitation, Faculty of Sport Sciences, Bu-Ali Sina University, Hamedan, Iran.; ^*c*^Department of Sport Injuries & Corrective Exercises, Faculty of Physical Education and Sport Sciences, ‎Tehran University, Tehran, Iran.

**Keywords:** Knee injury, KOS (Knee Outcome Survey), Football referees, Injury prevalence, Premier league, Injury mechanism

## Abstract

**Background:**

The purpose of this study was to determine the epidemiology and history of knee injury and its impact on activity limitation among football premier league professional referees in Iran.

**Methods:**

This was a descriptive study. 59 Football Premier League professional referees participated in the study. The knee injury related information such as injury history and mechanism was recorded. Injury related symptoms and their impacts on the activity limitation, ability to perform activities of daily living as well participation in sports and recreational activities was obtained through the Knee Outcome Survey (KOS).

**Results:**

The results indicated that 31 out of 59 participants reported the history of knee injury. In addition, 18.6%, 22.4% and 81% of the referees reported that they had been injured during the last 6 months of the last year, and at some point in their refereeing careers, respectively. Results further indicated that 48.8% of the injuries occurred in the non-dominant leg and they occurred more frequently during training sessions (52%). Furthermore, the value of KOS was 85 ± 13 for Activities of Daily Living subscale and 90 ± 9 for Sports and Recreational Activities subscale of the KOS.

**Conclusions:**

Knee injury was quite common among the Football Premier League professional referees. It was also indicated that the injuries occurred mainly due to insufficient physical fitness. Therefore, it is suggested that football referees undergo the proper warm-up program to avoid knee injury.

## Introduction

Football is considered to be the most popular sport in the world and approximately 270 million people (4%) of the world population are involved in it.^1-5^ Referees who make 5 million of this population play an important role in football. ^6,7^

Football referees perform the same activities such as walking and running during matches as the football players do.^8^ Although there are substantial differences between referees and players, they cover an average distance of 9 to 13 km at high intensity and even a longer distance is covered by professional referees.^9-11^ Football referees are quite active during matches and they are often older than football players. ^8^ Therefore, injuries of football referees are expected to be different from those of football players. ^12^

A number of studies have examined the anthropo-metric profiles of football referees, their movement pat-terns, their competencies, the quality and the level of their refereeing, their roles in refereeing (referee or assistant referee), the length of time they spend in train-ing and matches, and their psychological demands dur-ing matches and training.^10,13,14^

Bizzini and colleagues (2009) indicated that 39% of female referees were injured in Women’s World Cup in 2007, and that the incidence of injury was 1 per 20 matches (95% CI = 4.2-65.1).^13^ On average, 20.8% of injuries were recorded per 1000 match hours (95% CI = 4.17-31.7).^12,13^

Blake and colleagues (2009) showed that 61% of the referees were injured during 12 months (95% CI = 59-69), 56% of injury mechanisms were start-ups and fast short running, and 60% of injuries occurred at refer-eeing time.^15^

Gabrilo and colleagues (2013) indicated that over 40% of 342 male football referees were injured and over 60% of them reported musculoskeletal problems.^16^

A knee is the most important joint of a body in terms of stability, weight bearing, balance, mobility, and shock absorption during running. Bizzini and colleagues (2009) reported that one-third of referees underwent surgery due to musculoskeletal problems and knee operation is the most common surgery (20%).^17^ Furthermore, according to Mahdavi and Mirjani (2015), 57.1% of all knee injuries were related to the dominant leg and injuries of an anterior cruciate ligament (ACL) was the most prevalent (66%).^18^

Since referees are an integral part of the game of football and their absence can cause various problems, optimum health is a major factor for their perfect refereeing.^16^ The most common type of injury among football referees is the knee injury.^17,18^ The sufficient recovery time of this injury ranges from at least 6 weeks to 6 months^19^ which means a referee has to be away from refereeing from 6 weeks to 6 months. Therefore, knee injuries are highly important in football referees. The previous studies have mainly focused on the prevalence of injuries and few of them have investigated the prevention of the injuries among football referees.^20^ Besides, no research study has ever been conducted to examine knee injuries of football referees using the Knee Outcome Survey (KOS). 

This study aimed to investigate the epidemiology and history of knee injury and its impact on activity limitation among football premier league professional referees. 

## Methods 

**Study Design**

This study was cross-sectional.

**Subjects**

The study sample was composed of 59 football referees with grade 1, 2 and international-level officiating in the Iranian Premier League.

**Procedure**

One of the researchers filled out the questionnaires in person through face-to-face interviews with the referees at the Football Hotel in February 2016 when they were going to undergo assessment. They were asked to answer the questions accurately and ensured confidentiality of all information collected.

**Measurements**

The following questionnaires were used to collect the needed information:

**1. Personal Information Questionnaire:**height, weight, age, education level, and Body Mass Index.

**2. Sports Information Questionnaire:**sports history, number of training sessions, number of days and hours of training, training duration, type and duration of warm-up.

**3. Knee Injury History Questionnaire:**injury history, the injured leg (dominant or non-dominant), mechanism of injury, time of injury (in matches or training sessions), type of therapy, and special care of the injury.

**4. Knee outcome survey:**the KOS which is a patient-completed questionnaire was used to determine symptoms, functional limitation, and disability of the knee joint resulting from various knee injuries during activities of daily living and sports.^21^

The KOS is used for both athletes and elderly people,^22,23^ and investigates various injuries, including knee ligament injuries, meniscus tears, meniscal cartilage lesions, patellofemoral pain syndrome, dislocation of the knee, and osteoarthritis.^21,24^ The KOS has 2 subscales consisting of Activities of Daily Living Scale (ADLS) and Sports Activity Scale (SAS). Knee-Rating Scale has demonstrated high reliability and validity with the KOS subscales (0.97, 0.97, respectively for SAS; 0.78, 0.97, respectively for ADLS).^20,21,24^

The ADLS is a 14-item scale for activities of daily living. Six items assess the effects of knee symptoms such as pain, stiffness, swelling, buckling, ‎weakness, and limping on ability to perform activities of daily living, and 8 items assess the ‎effects of knee condition on the ability to perform specific functional tasks such as going up and ‎down the stairs, standing, kneeling, squatting, sitting with the knee bent, and rising from a ‎chair. 

The SAS is an 11-item scale which assesses the effects of knee symptoms on the ability to perform sports and recreational activities (7 items) and the effects of knee condition on the ability to perform specific skills such as straight running, jumping and landing, cutting and pivoting, quick stopping and starting (4 items). 

Each item is rated on a 5-point scale. The score can range from 0 to 70 for the ADLS and from 0 to 55 for the SAS. The overall ADLS and the SAS percent rating were calculated and presented.^21^ Lower percentages reflect higher levels of disability.

**Statistical Analysis**

Descriptive statistics were reported as frequency, mean, and standard deviation. They were used to determine the injury prevalence and characteristics of the sample. Pearson Correlation Coefficient was used to measure the relationships between the SAS and the ADLS, and the current level of self-reported knee joint function with both the SAS and the ADLS to evaluate the proprioception in the participants. An alpha level of p<0.05 was used to establish statistical significance. The statistical analyses were performed using SPSS version 22. 

## Results

Anthropometric characteristics and sports information of the participants are presented in Table 1.

**Table 1:** Sociodemographic and sports information of Iran’ Football Premier League professional referees (N=59).

Variables	Frequency or (mean)	Percentage	SD	Range	Confidence Interval (CLs)
**Statistical characteristics**					
Age	36.30		4.1	28 - 44	37.42 – 35.19
Weight	74.55		6.3	63 - 90	76.25 - 72.86
Height	179		5.8	167 - 193	180.68 – 177.57
BMI	23.3		1.6	18 - 29	23.75 – 22.87
**Education level**					
Doctoral degree	2				
Master’s degree	25				
Bachelor’s degree	32				
**Sports Information**					
**Training days per week**					
1-2 days	2	3.4			
2-3 days	19	32.2			
3-4 days	26	44.1			
5 ≤	12	20.3			
*Training sessions per week					
≤ 3 sessions	18	30.5			
4-5 sessions	27	45.8			
5-6 sessions	9	15.3			
6 sessions ≤	5	8.5			
**Training sessions per day**					
1 session	46	78			
2 sessions	6	10.2			
3 sessions ≤	7	11.9			
**Length of training per session**					
≤ 1 hour	6	10.2			
1-2 hours	49	83.1			
2-3 hours	4	6.8			
**Length of warm-up per session**					
≤ 15 minutes	6	10.2			
15-30 minutes	41	69.5			
31-45 minutes	1	1.7			
**Type of warm-up**					
Stretching exercises	3	5.1			
running	10	16.9			
Sports specialized training	12	20.3			
Stretching exercises and running	21	35.6			
Sports specialized training and Stretching exercises	1	1.7			
Sports specialized training, Stretching exercises, and running	12	20.3			

*For example odd days

31 out of 59 participants reported the history of knee injury. 18.6%, 22.4% and 81% of the referees reported that they had been injured during the last 6 months of the last year, and at some point in their refereeing careers, respectively.

48.8% of the injuries occurred in the non-dominant leg and they occurred more frequently during training sessions (52%).

The characteristics, type, and mechanisms of the referees’ knee injuries are presented in Table 2.

**Table 2:** Knee injury information.

Variables	Related Question	Frequency	Percentage
**Injury incidence time**	Match	12	42
	Training	15	52
	Both	3	10
**Injury type**	Anterior cruciate ligament	3	9.1
	Cruciate ligament posterior	1	2
	Medial collateral ligament	4	9.1
	Lateral collateral ligament	4	9.1
	Medial or lateral meniscus	18	40
	Patella ligament injury	2	4
	Articular cartilage injury	5	11
	Anterior knee pain	6	13
**Injury mechanism**	Landing	5	14
	Blowing	8	22
	Pivoting	16	74.1
	Falling	5	5
	Sudden stop	3	8
**Injury treatment**	No treatment	2	3
	Self-treatment (ice, heat…)	15	28
	Physiotherapy	24	42
	Medication	5	9
	Orthopedic cast or splint	4	7
	Surgery	5	7.5
**Time away from sport**	1-7 days	11	36.7
	8-20 days	10	33.3
	<21 days	7	30

The total scores were 85±13 and 90±9 for the ADLS and the SAS, respectively. In addition, the referees’ current level (at the time of the study) of self-reported knee join function were 79 ± 14 and 82±12 for the ADLS and the SAS, respectively. 

Furthermore, the correlation coefficient of the ADLS and the SAS with the referees’ self-reported knee functions were 0.47 (p =0.01) and 0.63 (p =0.001), respectively. Table 3 and 4 present more information on the ADLS and the SAS.

**Table 3:** Effects of knee symptoms on the ability to perform ADLS and performing SAS.

Variables	No symptom		No effects on activities		Slight effects on Activities		Moderate effects on activities		Sever effects on activities		Preventing activities	
	f	%	f	%	f	%	f	%	f	%	f	%
**Activities of Daily Living**												
Pain	17	56.7	6	20	7	23.3	-	-	-	-	-	-
Stiffness	15	50	8	26.7	5	16.7	2	6.7	-	-	-	-
Swelling	19	63.3	6	20	4	13.3	1	3.3	-	-	-	-
Giving way, buckling, or shift-ing of the knee	20	66.7	5	16.7	4	13.3	1	3.3	-	-	-	-
Weakness	13	43.3	8	26.7	5	16.7	4	13.3	-	-	-	-
Limping	18	60	10	33.3	-	-	1	3.3	1	3.3	-	-
**Sports and Recreational Activities**												
Pain	21	70	6	20	2	6.7	1	3.3	-	-	-	-
Grinding or grating	22	76.3	6	20	2	6.7	-	-	-	-	-	-
Stiffness	18	60	11	36.7	1	3.3	-	-	-	-	-	-
Swelling	25	86.2	3	10	1	3.3	-	-	-	-	-	-
Slipping or partial giving way of knee	20	66.7	8	26.7	2	6.7	-	-	-	-	-	-
Buckling or full giving way of knee	24	80	5	16.7	1	3.3	-	-	-	-	-	-
Weakness	21	70	8	26.7	1	3.3	-	-	-	-	-	-

**Table 4:** Functional limitations with ADLS and SAS.

Variables	No difficult at all		Minimally difficult		Somewhat difficult		Fairly difficult		Very difficult		Unable to do	
	f	%	f	%	f	%	f	%	f	%	f	%
**Activities of Daily Living**												
Walk	24	80	5	16.7	1	3.3	-	-	-	-	-	-
Go up stairs	16	53.3	9	30	3	10	2	6.7	-	-	-	-
Go down stairs	18	60	9	30	3	10	-	-	-	-	-	-
Stand	20	66.7	9	30	1	3.3	-	-	-	-	-	-
Kneel on front of your knee	14	46.7	8	13.6	4	13.3	3	10	1	3.3	-	-
Squat	11	36.7	12	40	5	16.7	1	3.3	1	3.3	-	-
Sit with your knee bent	8	26.7	5	16.7	10	33.3	4	13.3	3	10	-	-
Rise from a chair	21	70	8	26.7	1	3.3	-	-	-	-	-	-
**Sports and Recreational Activities**												
Run straight ahead	24	80	5	16.7	1	3.3	-	-	-	-	-	-
Jump and land on your involved leg	15	50	13	43.3	2	6.7	-	-	-	-	-	-
Stop and start quickly	16	53.3	10	33.3	3	10	1	3.3	-	-	-	-
Cut and pivot on the involved leg	11	36.7	15	50	4	13.3	-	-	-	-	-	-

## Discussion

This study aimed to investigate the prevalence and the mechanism of a knee injury and to identify the effects of knee injuries on knee function in the activities of daily living, sports activities, and proprioception of the Football Premier League professional referees in Iran. The study indicated that 81% of the referees had suffered knee injuries. A function of muscles surrounding knee ‎helps the knee movements and stabilization. Performance of these muscles may be affected by fatigue during the training sessions as the fatigued muscles can’t generate appropriate joint stability which in turn results in knee injuries.^25-27^ These findings are in line with the results by Bizzini and colleagues (2009) and Paes (2011).^12,28^ More focus on aerobic activities (46.7% CI = 41.1-52.3) and limited attention to warm-up and body flexibility (17.8 CI = 13.3-23.4) are considered the main causes of injuries.^29^ Referees should be involved in relatively higher levels of activities than those in matches. ^30^ These kinds of activities are called high-frequency training. ^31^

The results showed that knee injuries occurred more frequently during the training sessions (52%) which can be due to the duration of training. Referees practice for 1-2 hours per session (83.1%) and 4-3 days per week (44.1%). It also seems that the duration of training is longer for professional referees than for beginners.^17^ This volume of practice increases mechanical tension on the lower extremities, leading to an overall feeling of tiredness or lack of energy, thus increasing the risk of injury.^25^ The statistics show that the levels of physical fitness in more than half of Iranian football referees are rather lower than those of football referees in other countries. Thus, Iranian football referees should focus more on strength and conditioning training to prevent injuries. These findings are in agreement with the results by Bizzini and colleagues (2009) and Silva (2014),^32^ but not with the findings of Blake and colleagues (2009), Wilson and colleagues (2011) and Mahdavi and Mirjani (2015).

The results have revealed that meniscus injuries are the most prevalent type of knee injuries owing to knee rotation (74.1%). Meniscus injuries occur due to a combination of compression force and too much rotation while pivoting. In this case, the meniscus collagen tissue cannot endure the force leading to meniscus tear.^33^ Referees’ activities also include frequent pivoting, rotation and changing direction. In addition, tired referees are prone to knee joint laxity which in turn could increase valgus/varus stress on ‎knee joint, thus resulting in injury.^25,27^ ‎These findings are in line with those by Bizzini and colleagues (2009). 

The results also indicated that the Football Premier League professional referees resumed refereeing after a week of injury (36.7%). These findings are consistent with those by Wilson and colleagues (2011) but not with those by Bizzini and colleagues (2009) and Mahdavi and Mirjani (2015). The importance of refereeing in football can account for the rapid resumption of refereeing by a referee. Insufficient recovery can lead to re-injury which endangers referees’ health. Since referees’ health and refereeing are directly related, the quality of refereeing is also affected. 

The study showed that the most frequently used therapy was physiotherapy which confirms the findings of Mahdavi and Mirjani (2015).

The results from Knee-Rating Scale and the score assigned by the referees to their knee function in both the ADLS and the SAS are indicative of the referees’ high level of proprioception in their knee joint after refereeing resumption. According to Adachi and colleague (2002) knee joint instability does not affect proprioceptive function of the knee.^34^ Moreover, Good and colleagues (1999) stated that knee position sense did not differ for injured and non-injured knees. This finding may have been due to measurement error. Also, no exact measurement method had ever been designed which means the issue needs to be further investigated.^35^ However, Skinner and Barrack (1991) mentioned that weakness in knee joint proprioception is an effective factor in the etiology of meniscus lesions and can lead to degenerative joint disease.^36^

Knee injuries caused relatively more disorders in the activities of daily living than sports activities. The most frequent complaint was about sitting with the knees bent (Table 4). The knee injuries seem to be mostly related to meniscus which consequently lead to the reduction of knee joint range of motion.^37,38^ This is the first time that the KOS has been applied in football referees. Thus, there is no similar information to be compared with the obtained results. 

Based on the results of this study, it is concluded that the knee of the non-dominant leg is more prone to injuries. The non-dominant leg is usually weaker than dominant leg because the non-dominant leg plays the role of a supporter and a stabilizer in most movements and sports activities. Thus, non-dominant leg would tolerate more pressure which makes it more susceptible to injury.^39,40^ This can be reduced by appropriate strength training program and improvement of muscle balance in agonist and antagonist muscles (hamstrings and quadriceps). 

The Premier League referees were mostly injured during training sessions which can be attributed to increases in the training volume and a greater focus on aerobic exercises. It is proposed that training volume should be adjusted according to training seasons. Moreover, Interval Training should predominate.^30^ Besides, referees usually pay more attention to basic stretching and running (35.6%) which is considered to be an important factor causing injuries.^12^

This study has some limitations which have to be pointed out. First, the KOS is a self-reported scale. Therefore, the results may have been affected by recall bias. Second, we did not collect data related to the knee injuries of female football referees. Future research should focus on female football referees to see if the prevalence and mechanism of their injuries are different from those of male football referees. Third, the authors could not afford any more time to use objective assessment tools such as Functional Movement Screening (FMS) to see if they can predict injury. It is recommended that other functional assessment tests should be incorporated in addition to the self-reported questionnaires. 

The findings of this study can be used by the Referees Committee of the Football Federation Islamic Republic of Iran, football referees and coaches to design and apply specific warm-up program for football referees in order to prevent injuries and reduce the costs and time loss by referees.

It is recommended that special exercises such as proprioceptive, strength-training (e.g. Nordic hamstring), flexibility, and endurance exercises should be included in warm-up routine to prevent knee injuries.

## Conclusion

Knee injury was quite common among the Football Premier League professional referees. According to the results, the injuries occurred mainly due to insufficient physical fitness. Based on the background of the study, football referees are more active than football players and they run over longer distances. Therefore, football referees should undergo similar training to football players in order to prevent injuries.

**Acknowledgements**

We gratefully acknowledge the Football Premier League professional referees who participated in the present study. We would also like to appreciate the head of the Referees Committee of the Football Federation Islamic Republic of Iran and all other personnel contributing to the implementation of this study. 
